# Structure-based drug discovery for combating influenza virus by targeting the PA–PB1 interaction

**DOI:** 10.1038/s41598-017-10021-w

**Published:** 2017-08-25

**Authors:** Ken Watanabe, Takeshi Ishikawa, Hiroki Otaki, Satoshi Mizuta, Tsuyoshi Hamada, Takehiro Nakagaki, Daisuke Ishibashi, Shuzo Urata, Jiro Yasuda, Yoshimasa Tanaka, Noriyuki Nishida

**Affiliations:** 1 Department of Molecular Microbiology and Immunology, Graduate School of Biomedical Sciences, Nagasaki University, 1-12-4 Sakamoto, Nagasaki, 852-8523 Japan; 2 Nagasaki Advanced Computing Center, Nagasaki University, 1-14 Bunkyo-machi, Nagasaki, 852-8521 Japan; 3 Department of Emerging Infectious Diseases, Institute of Tropical Medicine, Nagasaki University, 1-12-4 Sakamoto, Nagasaki, 852-8523 Japan

## Abstract

Influenza virus infections are serious public health concerns throughout the world. The development of compounds with novel mechanisms of action is urgently required due to the emergence of viruses with resistance to the currently-approved anti-influenza viral drugs. We performed *in silico* screening using a structure-based drug discovery algorithm called Nagasaki University Docking Engine (NUDE), which is optimised for a GPU-based supercomputer (DEstination for Gpu Intensive MAchine; DEGIMA), by targeting influenza viral PA protein. The compounds selected by NUDE were tested for anti-influenza virus activity using a cell-based assay. The most potent compound, designated as PA-49, is a medium-sized quinolinone derivative bearing a tetrazole moiety, and it inhibited the replication of influenza virus A/WSN/33 at a half maximal inhibitory concentration of 0.47 μM. PA-49 has the ability to bind PA and its anti-influenza activity was promising against various influenza strains, including a clinical isolate of A(H1N1)pdm09 and type B viruses. The docking simulation suggested that PA-49 interrupts the PA–PB1 interface where important amino acids are mostly conserved in the virus strains tested, suggesting the strain independent utility. Because our NUDE/DEGIMA system is rapid and efficient, it may help effective drug discovery against the influenza virus and other emerging viruses.

## Introduction

Influenza is caused by acute influenza virus infection. Influenza pandemics occur due to antigenic shifts in the virus and they have caused significant morbidity and mortality in humans. At present, two classes of anti-influenza viral medicines have been approved: M2 inhibitors (amantadine and rimantadine) and neuraminidase (NA) inhibitors, such as, oseltamivir^1^. However, influenza viruses have mutated to become resistant to these inhibitors, thereby reducing their efficacy^2^. For example, oseltamivir-resistant seasonal H1N1 virus spread worldwide very rapidly during the 2007/2008 influenza season^3–5^. To avoid emergence of drug-resistant viruses, the combined use of antiviral drugs with different mechanisms of action is a good strategy as has been reported in the treatment of human immunodeficiency virus (HIV)-1. Therefore, the development of anti-influenza viral drugs with novel mechanism of action is needed.

Influenza A and B viruses are enveloped, negative-stranded RNA viruses with eight-segmented genomes. The influenza virus life cycle initiates by binding to the cell surface via hemagglutinin (HA), which is followed by fusion of the viral membrane and endosome. The viral ribonucleoprotein complex (vRNP) will be released into the cytoplasm by function of M2 protein of the virus. vRNP comprises viral RNA (vRNA), nucleoprotein (NP) and the three subunits of viral RNA-dependent RNA polymerase (RdRp): PB1, PB2 and PA. vRNP is transported to the nucleus and early transcription and replication from vRNP occur with the assistance of host factors^6, 7^. The progeny vRNP is exported from the nucleus into the cytoplasm^8, 9^. Viral assembly and budding occur on the plasma membrane with the progeny vRNP and newly synthesised late proteins such as HA, NA, M1, M2 and NS2.

Among the 10 viral-encoded proteins, RdRp could be a good target for the development of new drugs^10^ because RdRp does not exist in mammals and is essential for replication. In addition, the amino acid residues in each RdRp subunit (PB1, PB2 and PA) are highly conserved among type A viruses. Of the three RdRp subunits, PB1 and PB2 are responsible for viral RNA synthesis and binding to the cap structure of cellular-capped RNA (required for the initiation of viral transcription), respectively, whereas PA has multiple roles, such as cleavage of the capped RNA^11^ and serine proteinase activities^12^. Lack of PA protein expression in a reverse genetics system led to no recovery of recombinant influenza virus^13^, thereby suggesting that PA has crucial roles in the virus life cycle^14^. PA interacts with PB1 and PB1 interacts with PB2 according to crystal structure analyses^15–17^. The C-terminus of PA (residues 239–716) interacts with the N-terminus (residues 1–25) of PB1. According to this model, the binding between PA and PB1 involves hydrogen bonds and hydrophobic contacts, where the N-terminal residues of PB1 are inserted in the pocket of PA^15^. To explore PA-targeting inhibitors, various techniques such as NMR method^18^, phylogenetic analysis^19^ and high-throughput ELISA-based screening^20, 21^ have been reported.

The crystal structure information accumulated for various proteins allows us to design new antivirals by structure-based drug design (SBDD). SBDD has been used widely for the development of antiviral drugs, such as the HIV-1 proteinase inhibitor, nelfinavir^22^ and influenza viral NA inhibitor zanamivir^23^. Recent advances in SBDD have largely been made due to the development of computer system and docking simulation software. To facilitate the identification of novel drugs, we recently developed the Nagasaki University Docking Engine (NUDE) which efficiently runs on the DEstination for Gpu Intensive MAchine (DEGIMA) supercomputer. Using this system, we have succeeded in development of anti-prion compounds^24^ and anti-influenza virus compounds that target influenza viral NP^25^. In the present study, we performed *in silico* screening of approximately 600,000 molecular structures with NUDE to identify compounds that can bind the pocket in PA bound by PB1. We found that a quinolinone derivative bearing a tetrazole moiety effectively suppressed the replication of influenza viruses.

## Results

### *In silico* and *in vitro* screening of anti-influenza virus compounds

**Figure 1:**
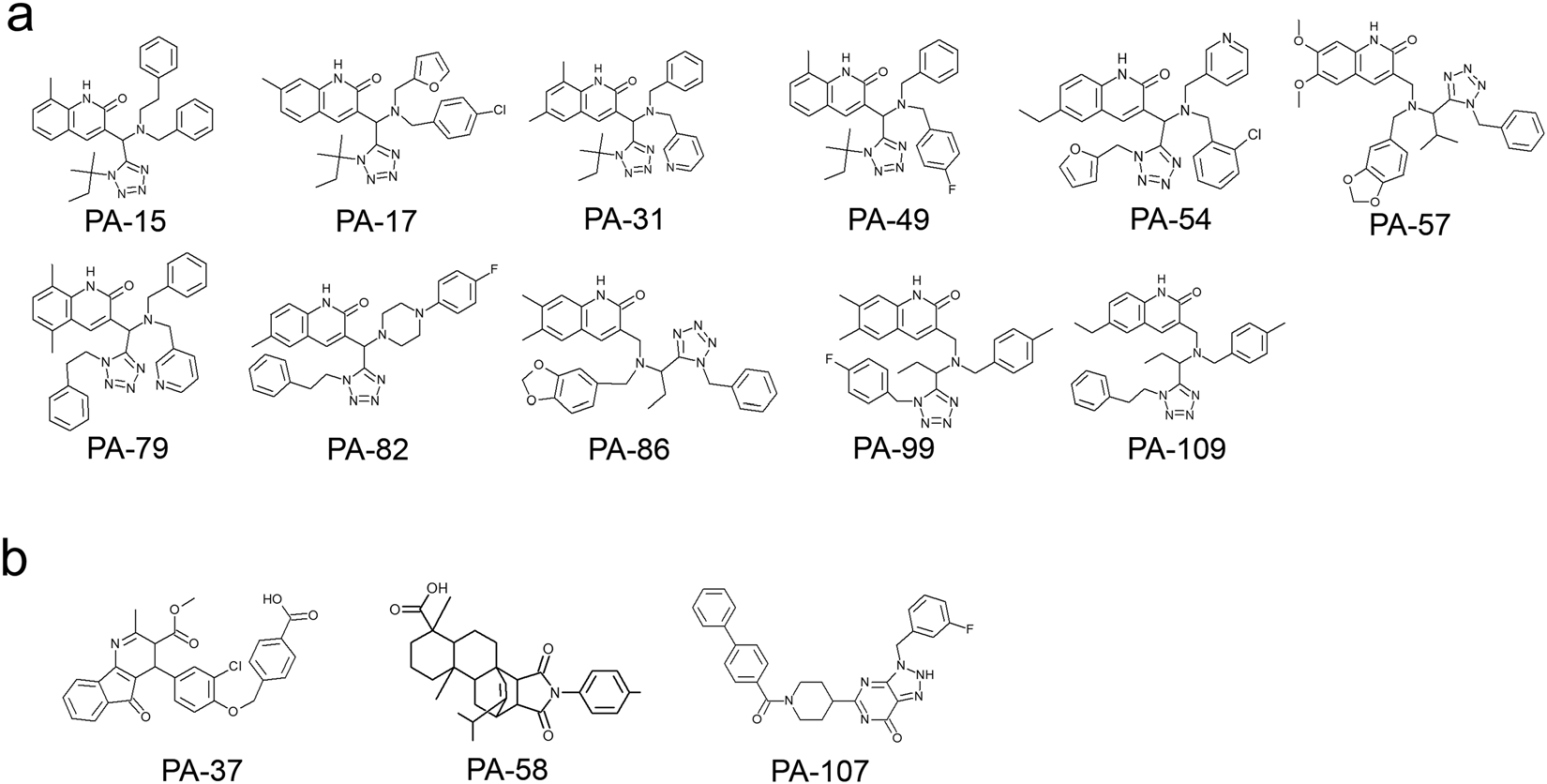
Hit compounds with anti-influenza virus activity. Hit compounds selected by *in silico* screening were tested to determine their anti-influenza virus activities via cell-based screening. Potent compounds with anti-influenza virus activities (MIC value < 20 μM) are shown. (**a**) Compounds bearing quinolinone and tetrazole moieties. (**b**) Compounds containing different scaffolds.

To obtain potent anti-influenza compounds we targeted the influenza viral polymerase subunit PA–PB1 interaction, which is essential for the viral life cycle^15^. Approximately 600,000 compounds in the chemical compound library were ranked based on the docking score obtained by the NUDE, and the top 136 compounds designated as PA-1 to PA-136 were selected as candidates of anti-influenza compounds. Among these compounds, 99 commercially available compounds were purchased and then subjected to cell-based screening using crystal violet (CV) assays, which reflected the virus infection-induced cytopathic effects (CPE) in cells (Supplementary Fig. S1). In this assay, when cells were infected with the virus, the cells detached from the bottom of the dish due to the appearance of CPE. We performed this assay using serially diluted compounds. The morphology of cells was observed under a microscope and the minimum inhibitory concentration (MIC) was estimated visually after CV staining. Fig. 1 summarises the structures of 14 compounds with MIC values less than 20 μM, where 11/14 compounds were quinolinone derivatives bearing a tetrazole moiety (Fig. 1a). PA-49 (ranking = 49^th^, docking score = −336.77) had the best MIC value of 1.7 μM but no significant cytotoxicity (Supplementary Fig. S2). Therefore, PA-49 was selected as representative of the quinolinone derivatives. Other different compounds (Fig. 1b) were also selected for further experiments (a 4-aza-9-fluorenone derivative (PA-37, MIC value = 1.7 μM, ranking = 37^th^, docking score = −339.311), tetracyclic compound (PA-58, MIC value = 16.6 μM, ranking = 58^th^, docking score = −336.051), and 8-azahypoxanthin derivative (PA-107, MIC value = 16.6 μM, ranking = 107^th^, docking score = −331.554)). These results suggest that our *in silico* programme, NUDE^24^, is a very useful tool for screening hit compounds.

### Evaluating the binding between hit compounds and PA protein using surface plasmon resonance (SPR) analysis

**Figure 2:**
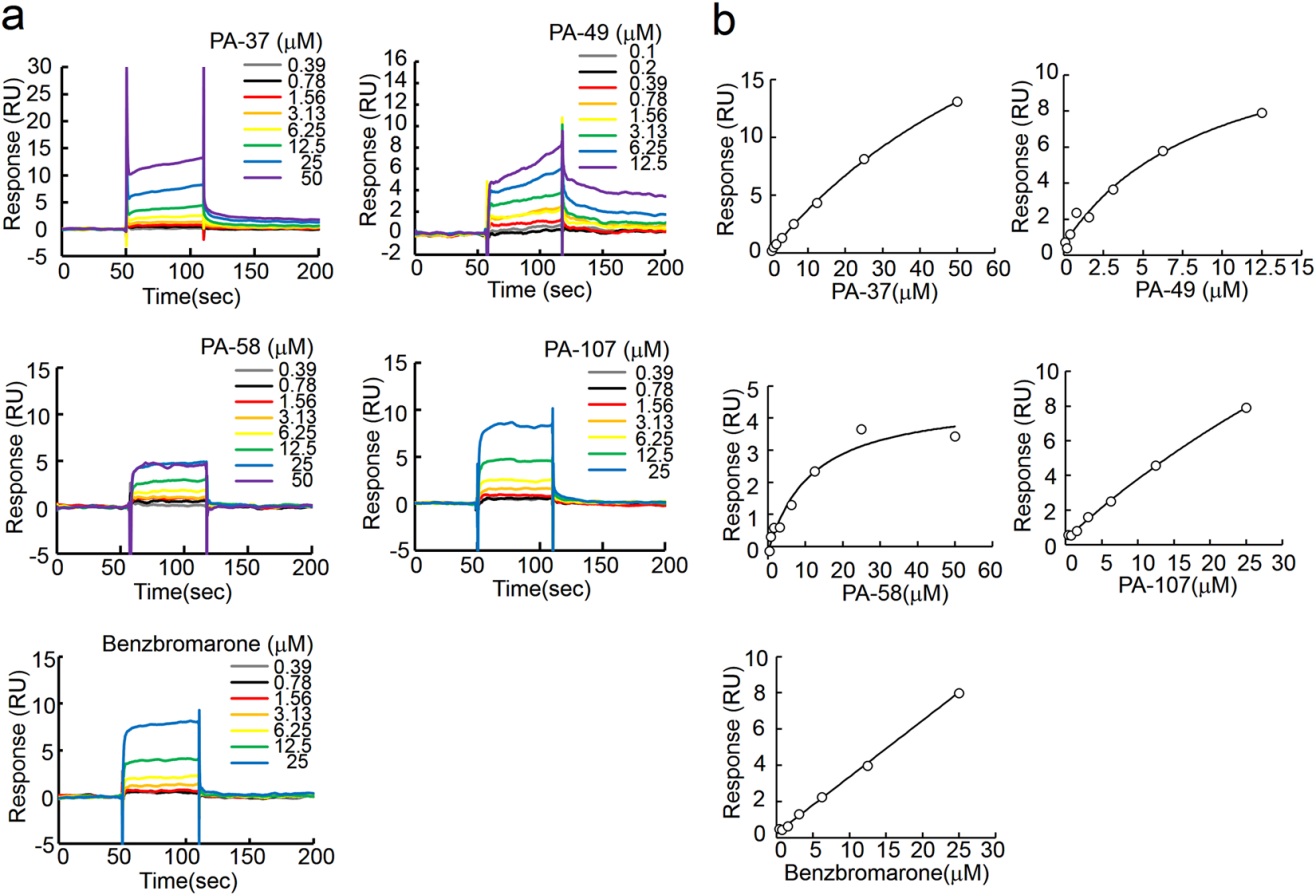
Direct binding between PA protein and hit compounds. Compounds selected by cell-based screening were subjected to surface plasmon resonance analysis. Anti-PA antibody was immobilised on the sensor chip and captured the recombinant PA protein before various concentrations of compounds were loaded as analytes. (**a**) Sensorgram and (**b**) fitting curves from the sensorgram were shown.

SPR analyses were performed to analyse the binding affinities of the hit compounds with PA protein (Fig. 2). Anti-PA antibody was immobilised on the sensor chip, and then captured approximately 2000 RU of the purified recombinant PA protein (residues 239–716). Signals obtained from the sensor chip-immobilised anti-PA antibody alone were subtracted as the background. Benzbromarone, a uricosuric agent, has been reported to bind the PB1-binding pocket of PA^26^, so we used benzbromarone as a positive control. In the presence of the compound the binding response was observed in a dose-dependent manner, suggesting that recombinant PA protein was suitable for evaluating the SPR analyses. PA-37, 49, 58 and 107 bound to PA protein in a dose-dependent manner (Fig. 2a). Using a 1:1 binding model, the dissociation constant (K_D_) was calculated from the fitting curve (Fig. 2b). The hit compounds had various K_D_ values (Table 1). PA-37 differed in terms of the MIC and K_D_ values, which suggests that PA-37 may have a target other than PA. PA-58 had good K_D_ values but significant toxicity (Supplementary Fig. S1). The binding affinity of PA-107 to PA was relatively weak. By contrast, PA-49 was the most potent compound with good MIC and K_D_ values of 1.7 μM and 7.5 μM, respectively. Therefore, we selected PA-49 for further analyses.

**Table 1:** Summary of anti-influenza viral activity of hit compounds.

compound	Cell-based screening	PA binding	Plaque inhibition assay IC_50_ (μM)^c^
	MIC (μM)^a^	K_D_ (μM)^b^	
PA-37	1.7	74.4 ± 27.0	N.D.^d^
PA-49	1.7	7.5 ± 2.0	0.47 ± 0.13
PA-58	16.6	13.6 ± 5.5	N.D.
PA-107	16.6	105 ± 24.8	N.D.

^a^MIC; minimum inhibitory concentration.

^b^Obtained from three independent SPR analyses using recombinant PA.

^c^IC_50_; half maximal inhibitory concentration.

^d^Not determined.

### PA-49 suppresses nuclear accumulation of PA

**Figure 3:**
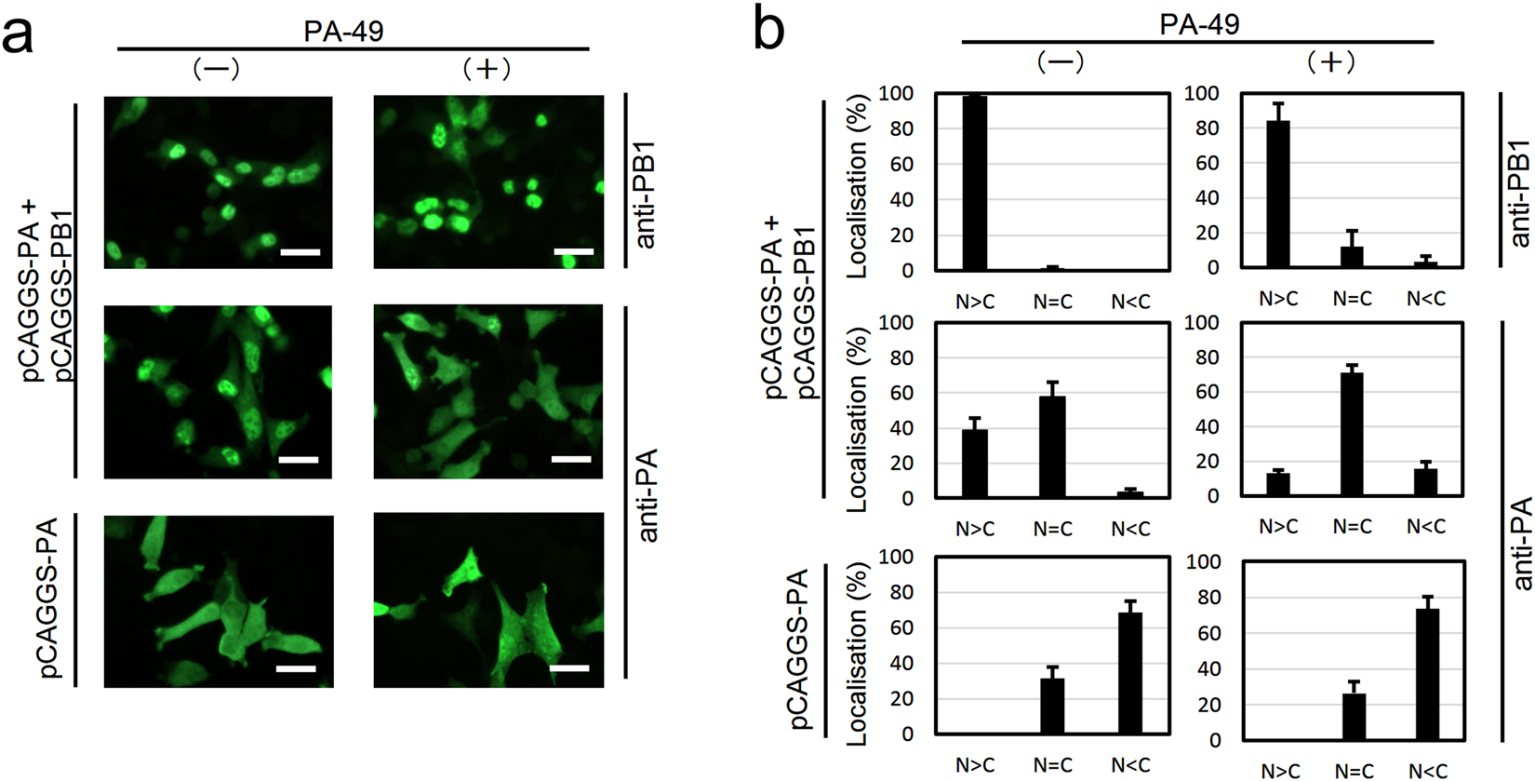
Effect of PA-49 on subcellular localisation of PA protein. In 24-well plates, HeLa cells were seeded on coverslips and grown to 70–80% confluence. Cells were then transfected with 0.5 μg of plasmid as indicated then 3.5 h after transfection, PA-49 was added then incubated further 25.5 h. Cells were fixed with 4% paraformaldehyde for 10 min, and were permeabilised by 0.1% NP40 in PBS for 20 min. The cells were incubated with 1% skim milk in PBS for 40 min. The cells were then reacted with anti-PA or -PB1 antibody. After washing, the cells were reacted with Alexa Flour 488-conjugated anti-rabbit IgG (Thermo Fisher Scientific K.K., Kanagawa Japan; A11008). (**a**) Subcellular localisation of PA and PB1 in the absence or the presence of 40 μM PA-49 were shown. Scale bar; 25 μm. (**b**) The percentage of cells showing greater nuclear than cytoplasmic localisation (N>C), nuclear equal to cytoplasmic localisation (N=C) or cytoplasmic localisation (N<C) was determined by direct counting. The mean and standard deviation obtained from three different areas were shown.

We performed a nuclear transportation-inhibition assay to confirm whether subcellular localisation of PA changes in the presence of PA-49 or not (Fig. 3 and Supplementary Fig. S3). It has been reported that the PB1 subunit is required for efficient nuclear accumulation of the PA subunit^27^. In Fig. 3, the experiment showed that in the absence of PA-49, PA dominantly localised in the nucleus where PB1 exists, and addition of PA-49 to PA- and PB1- cotransfected cells did change localisation of PA to the cytoplasm. These results suggest that PA-49 interfere with the PA–PB1 interaction.

### PA-49 suppresses influenza virus propagation

**Figure 4:**
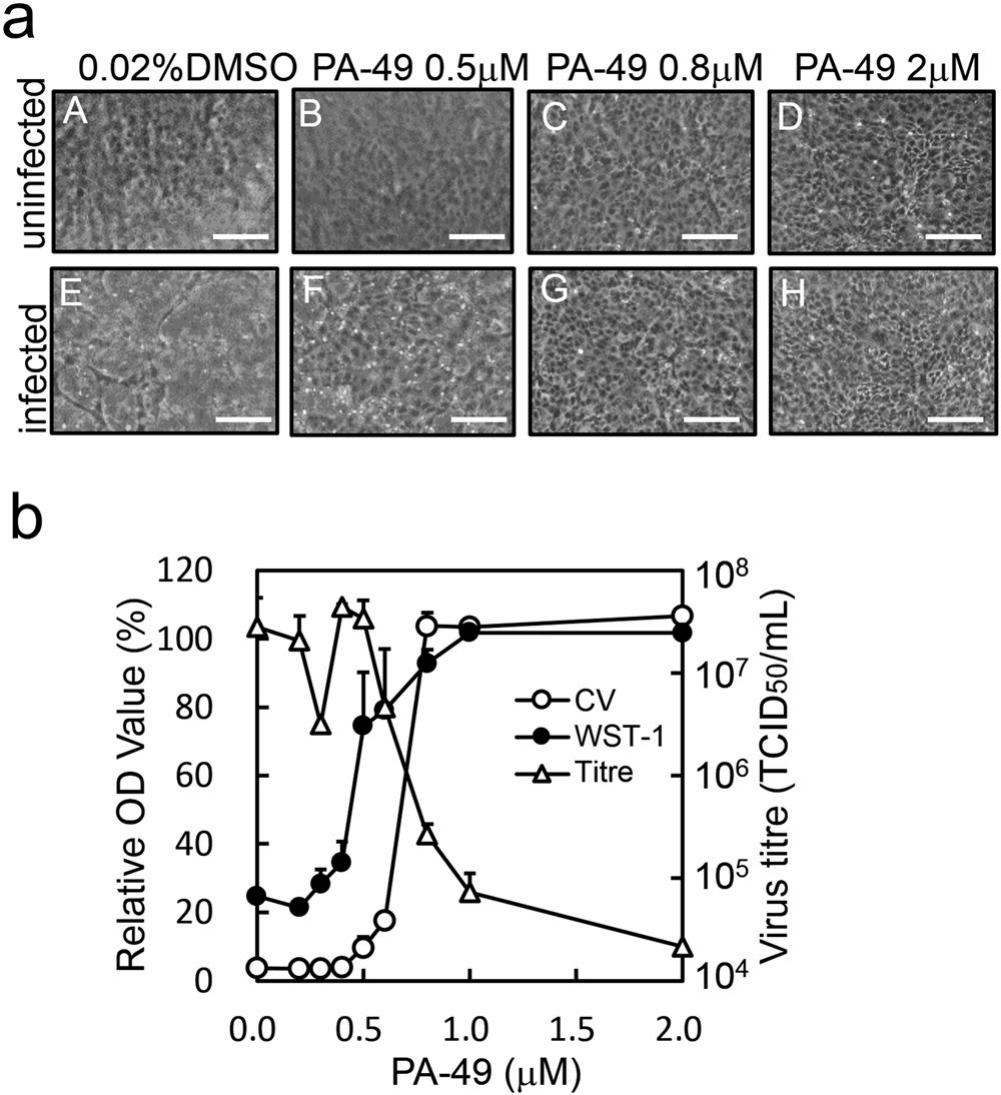
Anti-influenza virus activity of PA-49. The anti-influenza virus effects of PA-49 were evaluated as described in the Methods section. MDCK cells grown in 24-well plates were infected and treated with serial dilutions of PA-49. At 48 h after infection, the cell morphology (**a**) was observed (scale bar; 150 μm) and (**b**) the antiviral effects on cells were measured by the WST-1 assay (closed circles) or CV staining (open circles). Optical density (OD) values (%) are expressed relative to the percentage of cells without virus infection. Viral titres in the supernatant (open triangles) were measured by TCID_50_ assays.

We performed screening to select anti-influenza compounds based on CV assays (Supplementary Fig. S1) because CV staining is an alternative and rapid method for evaluating cytotoxicity and antiviral activities^28, 29^. To confirm the correlations between the CV assays and antiviral activities, we compared the antiviral activity of PA-49 based on cell morphology, water-soluble tetrazolium salt (WST-1), CV staining and 50% tissue culture infective dose (TCID_50_) assays (Fig. 4). When Madin–Darby canine kidney (MDCK) cells were infected with the influenza virus without PA-49, the cells detached from the bottom of the well (Fig. 4a, panel E). In the presence of 0.5 μM PA-49, the cells were partially attached (panel F), and the cell condition among the infected cells treated with 0.8 and 2 μM PA-49 are almost the same with uninfected control (panel A). The viral titre in the supernatant (Fig. 4b) was dose-dependent and it decreased dramatically (2.2 × 10^4^ TCID_50_/mL) by nearly 1/1000^th^ compared to the control after treatment with 2 μM PA-49 where the cell viability was good according to WST-1 measurements. The viral titres in the presence of 20 and 100 μM oseltamivir were measured as controls that were 2.1 × 10^5^ ± 1.5 × 10^5^ and 2.7 × 10^5^ ± 0.7 × 10^5^ TCID_50_/mL, respectively (Supplementary Fig. S4). These results suggest that PA-49 has potent antiviral activity. The viral titre appeared to be highly correlated with the relative optical density (OD) values determined in the CV assays. Therefore, we decided to use the CV assays for further evaluations of the 50% effective concentration (EC_50_) values.

### Determination of IC_50_ by plaque inhibition assay

**Figure 5:**
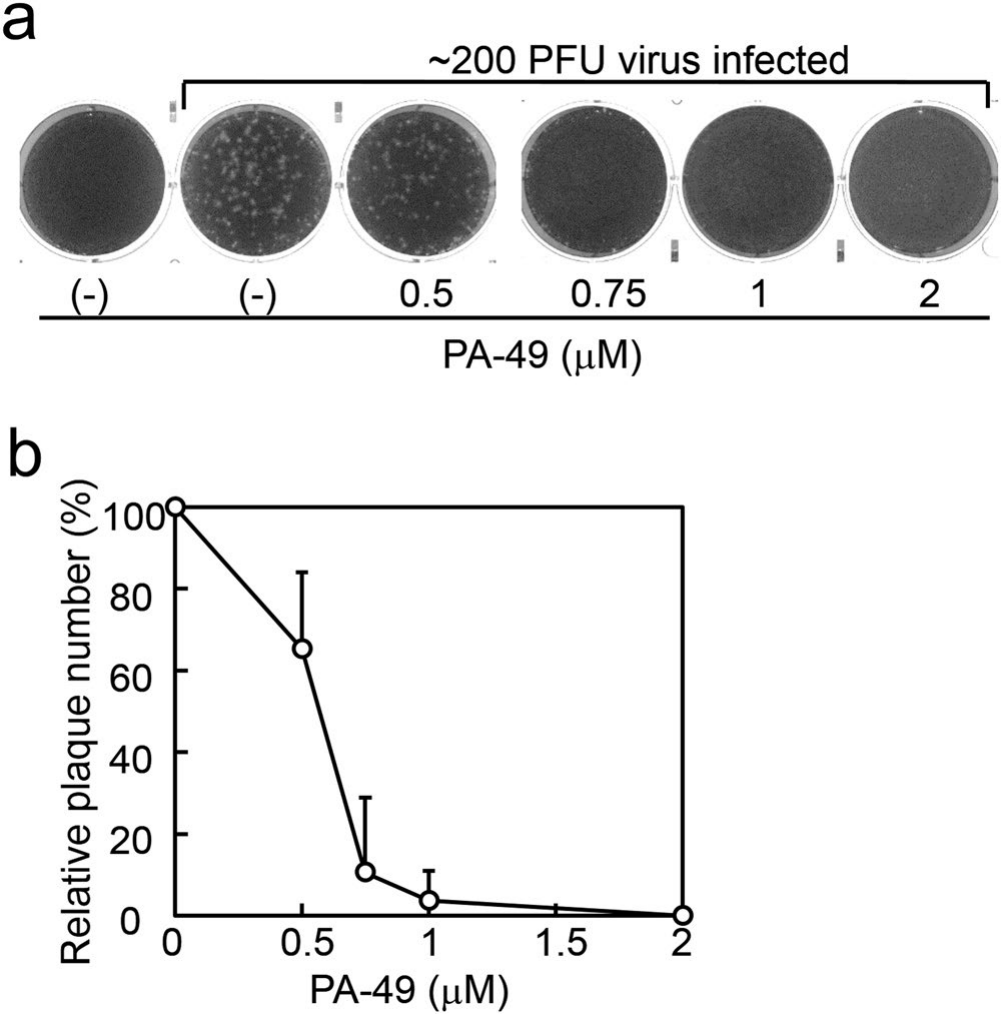
Determination of IC_50_ values by plaque inhibition assay. (**a**) Inhibition of plaque formation in the presence of PA-49. Confluent MDCK cells seeded in six-well plates (2 × 10^6^ cells/well) were then infected with approximately 200 pfu of A/WSN/33 virus at 37 °C for 1 h. After removing the medium, the cells were overlaid with 4 mL of PA-49-containing agarose solution (0.8% agarose, 0.1% BSA and MEM vitamin) and incubated for 2 days. The cells were fixed and stained with 0.5% Amido black. After washing with water and air drying, the number of plaques was counted visually. Representative results from three independent experiments are shown. (**b**) Results of plaque formation experiments in (**a**) and two other experiments are shown as relative plaque numbers.

Determining EC_50_ values using the CV assay is a feasible and rapid method for evaluating many samples. We also evaluated the antiviral activity of PA-49 using a plaque inhibition assay, which is the gold standard method for evaluating antiviral activity^30^, in order to determine whether plaque formation in MDCK cells was suppressed by PA-49 treatment. MDCK cells in six-well plates were infected with approximately 200 plaque-forming units (pfu) of virus and the cells were then overlaid with agarose solution containing various amounts of PA-49. The number of plaques formed decreased in a dose-dependent manner (Fig. 5a and b), where the 50% inhibitory concentration (IC_50_) determined by the plaque inhibition assay was 0.47 ± 0.13 μM (Table 1). These results suggest that PA-49 effectively suppressed influenza virus propagation in the cell culture.

### PA-49 suppresses the expression of viral proteins

**Figure 6:**
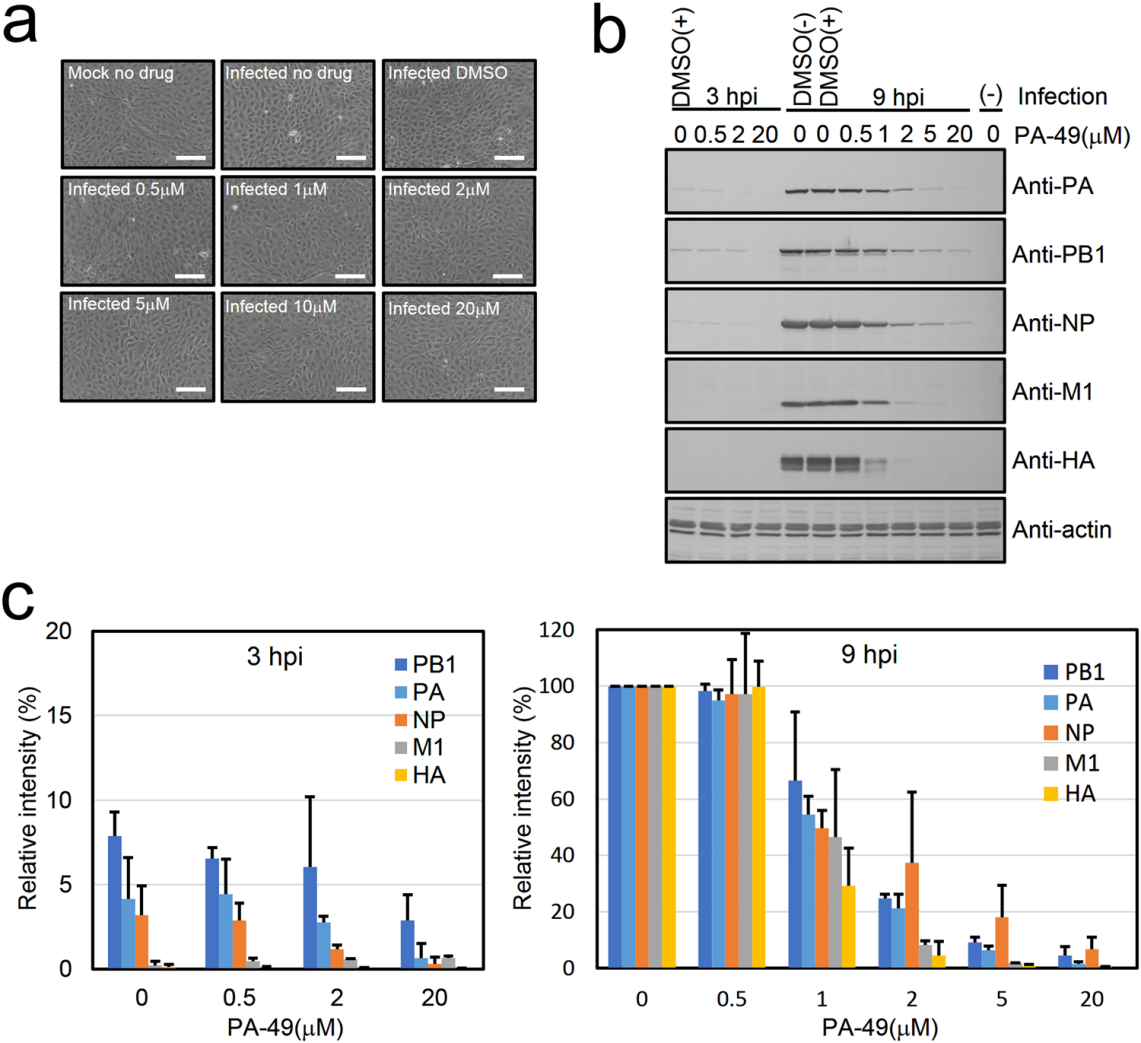
PA-49 suppresses the expression of viral proteins. MDCK cells (2 × 10^5^ cells/well) were seeded in 24-well plates and infected with A/WSN/33 at an MOI of 1 in the absence or presence of PA-49 (0.5–20 μM). (**a**) At 9 hpi, the cells were observed by phase contrast microscopy. Scale bar, 150 μm. (**b**) At 3 or 9 hpi, the cells were then lysed and subjected to 10% SDS-PAGE, and then transferred onto a polyvinylidene fluoride membrane. The membrane was incubated with a 1:30,000 dilution of anti-NP or anti-M1, 1:5,000 dilution of anti-HA or anti-PA, 1:3,000 dilution of anti-PB1, or 1:150 dilution of anti-actin for 4 h, followed by treatment with the biotinylated secondary antibody and streptavidin alkaline phosphatase, and visualisation using BCIP and NBP. (**c**) The band intensity was quantified by Image J software. The expression level of actin was used for normalisation. To calculate the relative intensity (%) for 3- and 9-h PA-49-treated samples, the band intensity of 9-h- infected cells without PA-49 treatment was shown as 100%. Data represent the means and standard deviation from two independent experiments.

The formation of the PA–PB1 complex is essential for obtaining a functional vRNP complex. In this study, we targeted the PB1-binding pocket of PA. Viral transcription/replication from the newly synthesized vRNP complex might not occur in the presence of PA-49. Thus, western blotting was performed to confirm the expression of viral proteins (Fig. 6). MDCK cells were infected at a multiplicity of infection (MOI) of 1 before adding increasing concentrations (0.5–20 μM) of PA-49. At 9 h post-infection (hpi), the control uninfected MDCK cells were attached tightly to the dish (Fig. 6a, mock no drug). The cell morphology and viability was not affected by the presence of different concentrations of PA-49 (0.5–20 μM), indicating the low toxicity of PA-49 (Supplementary Figs 5a and 6). The cells were recovered and analysed by western blotting (Fig. 6b and c). At 9 hpi, in the presence of PA-49, the band intensity was reduced in a dose-dependent manner. These results suggest that PA-49 suppresses influenza virus replication by inhibiting viral RNA synthesis.

### PA-49 affects virus replication but not virus particles, cells and adsorption

**Figure 7:**
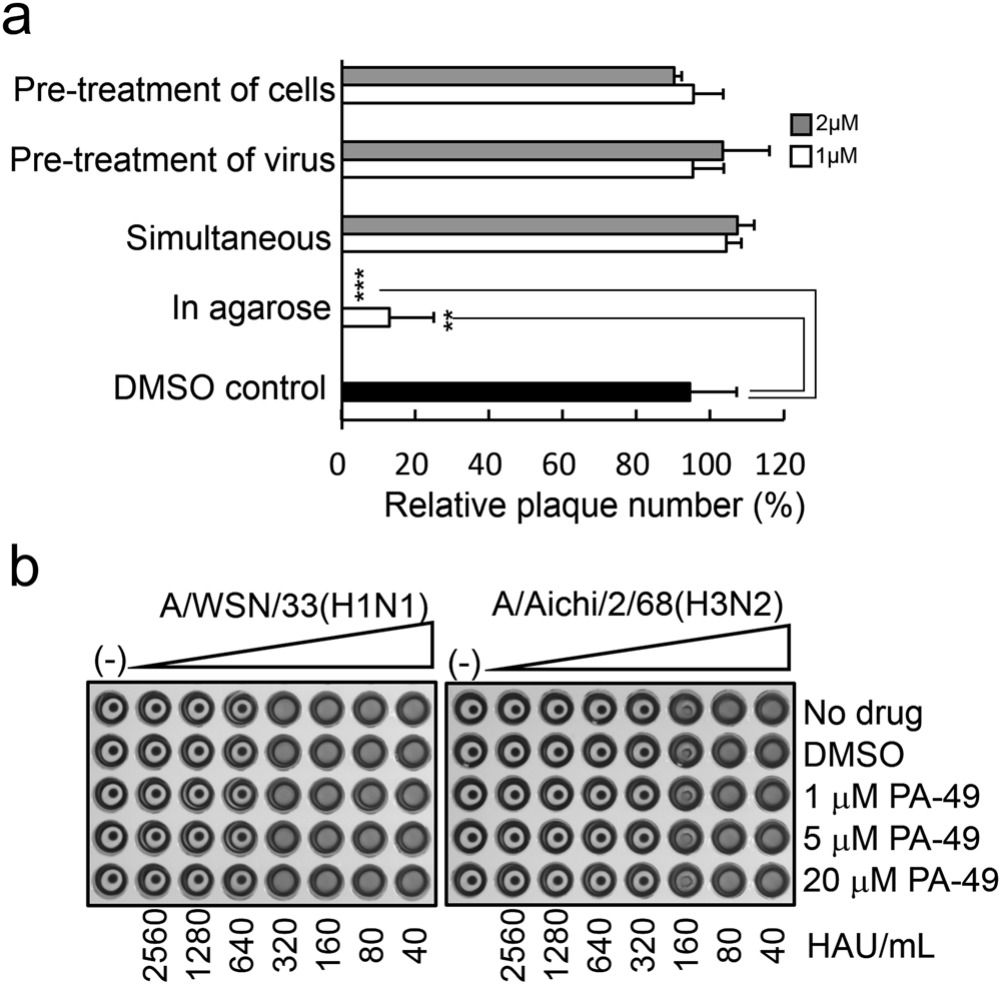
Mode of action of PA-49. (**a**) Time-of-addition experiments. Approximately 200 pfu of virus in MEM vitamin was used for infection. The detailed procedures for each treatment are as follows. (i) Pre-treatment of cells: before the plaque inhibition assays, MDCK cells were pre-treated with the test samples at 37 °C for 3 h. After removing the medium, cells were washed with MEM and infected by adding the viral suspension in MEM vitamin. (ii) Pre-treatment of virus: approximately 10^7^ pfu/mL of virus stock was pre-incubated with the test samples on ice for 3 h. The mixture was subsequently diluted in MEM vitamin and 400-μL aliquots (200 pfu) of the diluted mixture were used for infection. (iii) Simultaneous: 200-μL aliquots of the test samples in MEM vitamin were added to MDCK cells, followed by 200 μL of the virus suspension. The cells were then incubated for 1 h. (iv) In agarose: after viral infection for 1 h, the cells were overlaid with 4 mL of agarose solution containing the samples and MEM supplemented with 0.8% agarose, 0.1% BSA and 1% 100 × vitamin solution. Two days later, the cells were fixed and stained. Relative plaque numbers based on three independent experiments are shown. Grey and white bars show the 2 and 1 μM PA-49 treatments, respectively. **Indicates *p* < 0.01; ***indicates *p* < 0.001. (**b**) PA-49 does not inhibit the hemagglutination activity of viral HA protein. Twofold serial dilutions of A/WSN/33 and A/Aichi/2/68 in PBS, as well as 4, 20 and 80 μM of PA-49 in PBS were prepared. In 96-well plates, 25 μL of each serially diluted virus and PA-49 solution were mixed and then incubated at 4 °C for 1 h before adding 50 μL of 5% (v/v) chicken RBCs in PBS. Hemagglutination was observed after incubating at room temperature for 1 h.

PA-49 may inhibit the formation of viral polymerase PA–PB1 subunit, but PA-49 might also affect other parts of the virus life cycle, such as virus attachment or entry into cells. Thus, time-of-addition assays were performed to confirm the mode of action for PA-49 (Fig. 7a). The pre-treatment of cells and pre-treatment of virus with 1 and 2 μM PA-49 had no effects on the relative plaque numbers, thereby suggesting that PA-49 does not bind to a cellular receptor and that it lacks a virucidal activity. However, a significant reduction (over 99%) in the relative plaque number was observed when 2 μM of PA-49 was added to the agarose solution (‘in agarose’). Thus, PA-49 had significant antiviral activity against influenza virus when replication cycle occurs. These results were also supported by RT-PCR analysis (Supplementary Fig. S7). To exclude the possibility that PA-49 inhibits viral binding to the cellular receptor, a hemagglutination inhibition assay was performed using chicken red blood cells (RBCs) (Fig. 7b). As expected, hemagglutination occurred when RBCs were mixed with influenza virus (Fig. 7b, no drug), where the HA titres for A/WSN/33(H1N1) and A/Aichi/2/68(H3N2) were 320 and 160 HA units/mL, respectively. The presence of PA-49 (1–20 μM) did not affect the hemagglutination reaction. These results suggest that PA-49 has no effect during the binding/entry step of the infection process.

### Antiviral effect of PA-49 on various influenza viruses

Antiviral effect of PA-49 on various influenza viruses were tested using the CV assay (Table 2). The EC_50_ value of oseltamivir against A/WSN/33 was found to be similar to that reported previously^29^. When PA-49 was used in the analysis, the EC_50_ values against A/Virginia/ATCC2/2009, A/WSN/33, A/Puerto Rico/8/34 and A/Aichi/2/68, were similar, ranging from 0.53 to 0.62 μM. PA-49 was also effective against B/Lee/40 with an EC_50_ value of 0.92 μM. The viral titre in the supernatant was measured by the HA assay because PA-49 did not affect the hemagglutination of the H1N1 and H3N2 subtypes (Fig. 7b). The HA titres in the culture supernatants of A/WSN/33-, A/Puerto Rico/8/34- and A/Aichi/2/68- infected cells in the presence of 1 μM PA-49 were <0.8%, <0.8% and <3.1% compared with the no drug control, respectively. In addition, the IC_50_ and EC_50_ values determined for the A/WSN/33 strain were similar using the plaque inhibition and CV assays (compare Tables 1 and 2). We also tested the antiviral activity of PA-49 against several negative-stranded RNA viruses that have RdRp including severe fever with thrombocytopenia syndrome virus (SFTSV, *Bunyaviridae*), Hazara virus (*Bunyaviridae*), vesicular stomatitis virus (*Rhabdoviridae*) and lymphocytic choriomeningitis virus (*Arenaviridae*), because these RNA viruses have a similar replication mechanism with influenza virus^31, 32^. RdRp of these viruses is a single polypeptide called L protein, whereas that of influenza virus is composed of subunits of PA, PB1 and PB2. As expected, PA-49 did not show significant antiviral activity against these RNA viruses (Supplementary Fig. S5b). These results suggest that PA-49 can specifically inhibit the replication of influenza viruses types A and B.

**Table 2:** Efficacy of PA-49 against various strains of influenza virus.

Compound	CC_50_^a^ (μM)	Strain	Subtype	EC_50_^b^ (μM)	SI^c^	Relative titre^d^ (%)	
						1 μM	10 μM
Oseltamivir	>100	A/WSN/33	H1N1	4.0 ± 1.1	>24.8	14.6 ± 9.5	<1.6
PA-49	>100	A/WSN/33	H1N1	0.57 ± 0.02	>175	<0.8	<0.8
		A/Puerto Rico/8/34	H1N1	0.53 ± 0.01	>189	<0.8	<0.8
		A/Aichi/8/68	H3N2	0.56 ± 0.01	>179	<3.1	<3.1
		A/Virginia/ATCC2/2009^e^	H1N1	0.62 ± 0.02	>161	N/A^f^	N/A
		B/Lee/40	Type B	0.92 ± 0.07	>109	18.8 ± 8.8	9.4 ± 4.4

^a^CC_50_; 50% cytotoxic concentration estimated by CV assay.

^b^EC_50_; 50% effective concentration estimated by CV assay.

^c^SI: selective index = CC_50_/EC_50_.

^d^Relative virus titre in the presence of compound was estimated by HA assay *vs* no drug control.

^e^Clinical isolate of A(H1N1)pdm09 virus.

^f^This strain was not sensitive to chicken RBC.

## Discussion

To explore novel anti-influenza compounds using SBDD, focusing on the PA–PB1 interaction which co-crystal structure of PA and PB1 has also been reported^15^, we screened for compounds that targeted the major pocket in PA binding PB1 and inhibits the replication of influenza virus. We expanded our in-house library to approximately 600,000 compounds, and the top 99 compounds were then subjected to *in vitro* assays. Among that, the activity of PA-49 was promising against various influenza viruses, including types A and B (Table 2). Our plaque inhibition and CV assays suggested that PA-49 inhibits viral replication (Fig. 5 and Table 2). At 9 hpi, the expression levels of late viral proteins M1 and HA, which are synthesised from progeny vRNP, were effectively suppressed compared with those of early viral proteins PA, PB1 and NP, which are synthesised from incoming vRNP. PA-49 could suppress the PA–PB1 interaction (Fig. 3) when newly synthesised vRNP assembly occurred, whereas the incoming vRNP had already formed the PA–PB1 complex. The effect of PA-49 on the expression levels of viral proteins (Fig. 6b) was relatively weak compared with that on the viral titre in the supernatant (Fig. 4b), probably because the appropriate ratio of viral proteins must be expressed for efficient virion formation^13^.

**Figure 8:**
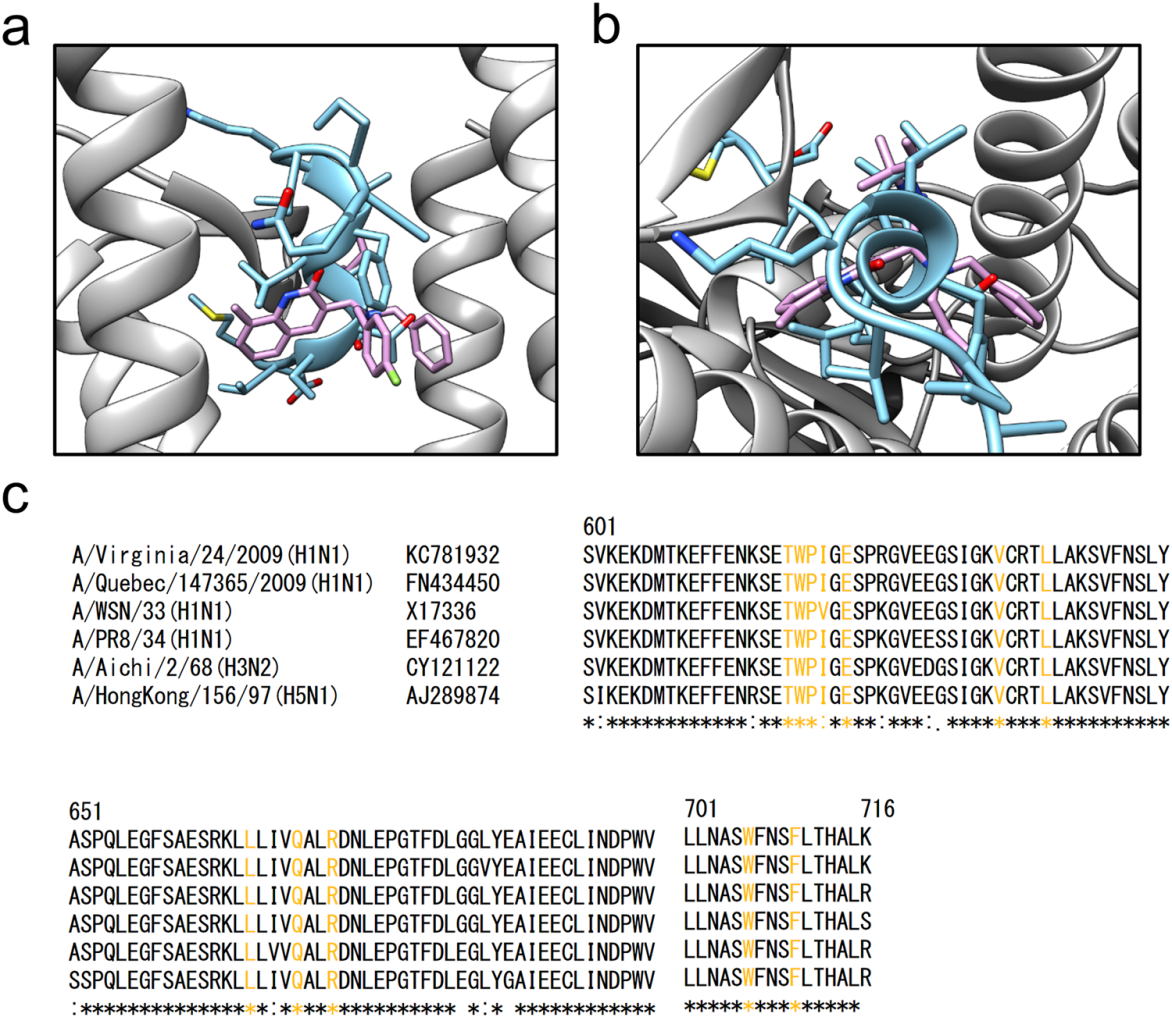
Binding mode of PA-49 compounds with influenza PA protein based on *in silico* calculations. (**a,b**) The crystal structure of the PA (grey)–PB1 (light blue) complex (PDB code: 2ZNL) and the binding structure of PA-49 from our docking simulations (pink) were overlaid: (**a**) side view and (**b**) top view. Side chains of PB1 protein are shown with stick representation. Hydrogen atoms are omitted for clarity. In order to obtain binding structure of PA–PA-49 complex, hydrogen atoms were added to the complex structure obtained from the docking simulation, followed by 1,000 steps of energy minimisation without any restraint using AMBER 10 software package^51^. For minimisation, AMBER ff99SB^52^ and GAFF^47^ force fields were used for the protein and ligand, respectively. Colour code for atom: N (blue), O (red), S (yellow) and F (light green). (**c**) Amino acid sequence alignment of influenza virus PA protein (residues 601–716) including H1N1, H3N2 and H5N1 subtypes. Residues that are required for PA–PB1 interface are labelled with yellow. Molecular structures in (**a**) and (**b**) were drawn using UCSF Chimera package^53^.

The EC_50_ values for PA-49 were similar against A/Virginia/ATCC2/2009(H1N1), A/WSN/33(H1N1), A/Puerto Rico/8/34(H1N1), A/Aichi/2/68(H3N2) and B/Lee/40 (Table 2), which suggests that PA-49 has a broad spectrum of anti-influenza virus activities. In general, the amino acid sequence of PA is highly conserved. It should be noted that the amino acid residues required to form the hydrogen bonds and hydrophobic contacts across the PA–PB1 interface^15^ are also conserved among various influenza A virus strains, including the H1N1, H3N2 and H5N1 subtypes (Fig. 8c). The docking simulation results suggested that PA-49 located in the centre of PB1-binding pocket of PA (Fig. 8a and b). The important amino acid residues for PA–PB1 interface are conserved in the virus strains tested (Fig. 8c), thereby suggesting the possible utility of PA-49 as an anti-influenza viral drug. To clarify the binding mode of PA-49, further analysis is needed based on structural analysis of the protein-compound complex by NMR and more accurate simulations including molecular dynamics simulation or fragment molecular orbital calculation.

Influenza A (H1N1)pdm09, H3N2 strain and two type B viruses are currently used for vaccine production, but it is likely that these viruses rapidly acquired resistance to oseltamivir and other NA inhibitors. The amino acid sequence of PA is also conserved in the oseltamivir-resistant clinical isolate of A(H1N1)pdm09 virus A/Quebec/147365/2009(H1N1) strain (Fig. 8c), which has an H275Y mutation in NA^33^, thereby suggesting that PA-49 may be effective against oseltamivir-resistant clinical isolates. Recently, we reported that a novel influenza virus inhibitor NUD-1 can suppress influenza virus replication by targeting the influenza virus NP^25^, but it does not inhibit the replication of type B viruses. By contrast, the inhibitory activity of PA-49 was observed against type B virus with EC_50_ values less than 1 μM (Table 2).

Interestingly, many quinolinone compounds like PA-49 were selected by our screening analysis. Quinolinone is a major scaffold and useful pharmacophore in many drugs^34^, such as the bronchodilator procaterol^35^, antithrombotic agent cilostazol^36^ and antipsychotic agents aripiprazole^37^. In addition, a similar monomethyl quinolinone with a tetrazole group has been reported as a novel tyrosine phosphatase inhibitor^38^. Eleven hit compounds with anti-influenza activity (MIC values less than 20 μM) had quinolinone moiety (Fig. 1). Other hit compounds that do not have this moiety (PA-37, 58 and 107) showed weak binding properties to PA protein (Table 1). The important role of this moiety on the binding to PA should be elucidated.

According to our results, among the 99 commercially available compounds that we screened, 88 compounds had molecular weights more than 500 (ranging from 303.2–596.7, median = 532.7). Because the PA cavity for PB1 binding is relatively wide (Fig. 8a and b), it is possible that a fairly large molecule is necessary to specifically inhibit (with high affinity) PB1 binding to the PA cavity. It has been suggested that lead compounds with molecular weights of less than 500 conform to Lipinski’s rule of five for drug-likeness^39^. Indeed, previously reported inhibitors targeting PA–PB1 interaction^26, 40, 41^ conform to this rule. However, recent advances in the development of protein–protein interaction inhibitors have demonstrated the suitability of medium-sized compounds with molecular weights of more than 500^42^. Recently, medium-sized compounds such as the antitumor agent eribulin (molecular weight = 729.9)^43^ and the BCL-2 inhibitor venetoclax (molecular weight = 868.4) for chronic lymphocytic leukaemia with 17p deletion^44^ have been approved.

In summary, we efficiently identified potent anti-influenza virus compounds that bind to PA by SBDD. PA-49 could be a lead compound in the development of novel anti-influenza drugs. Optimisation of the structure of PA-49 to increase its anti-influenza activity and *in vivo* experiments are currently in progress. Focusing on the inhibition of viral protein–protein interactions will facilitate the development of antiviral drugs and our strategy using NUDE/DEGIMA system may be applicable in emergent drug screening for many other fatal RNA viruses.

## Methods

### *In silico* screening

*In silico* screening used an original docking simulation programme NUDE, which was designed to run on a GPU system^24, 25^. The algorithm employed by NUDE is based on evolutionary Monte Carlo (EMC) techniques^45^ and an empirical free energy model is used to evaluate the binding energy of a ligand. We used an original chemical compound library comprising approximately 600,000 compounds. For each compound, a maximum of 50 conformations were generated using Open Babel software^46^, which were followed by energy minimisation with the GAFF force field^47^. In our docking simulation, the X-ray structure of influenza A virus PA was downloaded from the Protein Data Bank (PDB code: 2ZNL)^15^ as the receptor and a cubic space that included PB1-binding pocket of PA was employed as the search region. The docking simulation was performed using the NUDE/DEGIMA system.

### Chemicals, cells, viruses and antibodies

Hit compounds were purchased from Namiki Syoji Co. Ltd (Tokyo, Japan) and dissolved in 100% dimethyl sulfoxide (DMSO). Benzbromarone was purchased from Tokyo Chemical Industry Co. Ltd (Tokyo, Japan) and dissolved in 100% DMSO. Oseltamivir phosphate purchased from F. Hoffmann-La Roche Ltd (Basel, Switzerland) was dissolved in phosphate-buffered saline (PBS) at a concentration of 10 mM. All of the compounds were maintained at −30 °C until use. Before performing the experiments, the compounds were diluted with Eagle’s minimum essential medium (MEM) supplemented with 1% 100× vitamin solution (MEM vitamin). HeLa cells were obtained from Dr Takujiro Homma (Nagasaki University, Japan) and maintained in Dulbecco’s modified Eagle’s medium (Sigma Aldrich, St Louis, MO) containing 10% fetal bovine serum (FBS). MDCK cells kindly donated by Dr Kyosuke Nagata (Tsukuba University, Japan) were grown in MEM supplemented with 5% FBS. These cells were maintained at 37 °C in an atmosphere of 5% CO_2_. Influenza viruses were prepared as described previously^48^ and stored at −80 °C. Anti-HA (GTX127357), -M1 (GTX125928), -PA (GTX118991), -PB1 (GTX125923) and -NP (GTX125989) antibodies were purchased from GeneTex, Inc. (Irvine, CA). Anti-actin (A5060) antibody was purchased from Sigma Aldrich.

### Plasmid construction and purification of recombinant PA protein

To construct a plasmid encoding the His-tagged C-terminal portion of PA (residues 239–716), total RNA was prepared from A/WSN/33-infected MDCK cells. The RNA samples were reverse-transcribed using WSNPA1aa- pET15bNdeI (5′-CATATGGAAGATTTTGTGCGACAATGC-3′). PCR amplification was performed with the primer pair: WSNPA239aa- pET15bNdeI (5′-CATATGAACGGCTACATTGAGGGCAA-3′) and WSNPApET15bBamHIRev (5′-GGATCCCTATCTCAATGCATGTGTGAGGAA-3′). The PCR products were subcloned into T-vector pMD19. The resultant plasmid was digested with *Nde*I and *Bam*HI, and the fragment was subcloned into the *Nde*I-*Bam*HI site of pET15b. To express the PA_259–716_ protein, the pET15b-PA_259–716_ plasmid was transformed into BL21(DE3) pLysS. The protein was induced at 23 °C by adding 0.5 mM IPTG at an OD_600_ value of 0.7 for 5.5 h. The bacteria were disrupted by sonication in buffer containing 20 mM Tris-HCl (pH 7.9), 150 mM NaCl, 25 mM imidazole, 500 mM urea, 10 mM 2-mercaptoethanol and proteinase inhibitor cocktail (No. 03969-21, Nacalai Tesque), before purification using His60 Ni Superflow™ Resin (Takara Bio Inc, Shiga, Japan). His–PA_259–716_ was eluted with the same buffer containing 100–400 mM imidazole. The purified protein was dialysed in dialysis buffer containing 20 mM Tris-HCl (pH 7.9), 150 mM NaCl, 20% glycerol and 5 mM DTT, and then stored at −80 °C until use. To construct pCAGGS-PA and pCAGGS-PB1 plasmids, the WSN PA and PB1 genes were inserted into the multiple cloning site of pCAGGS vector, respectively.

### SPR analysis

Interactions between recombinant PA and compounds were evaluated by SPR using the Biacore T200 system (GE Healthcare UK Ltd, Buckinghamshire, UK), as described previously^49^ with some modifications. Briefly, we immobilised the anti-PA antibody on a CM5 sensor chip (GE Healthcare, BR-100530) using an amine coupling kit (GE Healthcare, BR-1000-50) and running buffer (10 mM HEPES pH 7.4, 150 mM NaCl and 0.1% Tween 20 [Sigma Aldrich]). The amount of immobilised antibodies was ~8,000 RU. Next, the recombinant PA protein were loaded at a flow rate of 30 μL/min for capture by the antibodies on the sensor chip. The amount of PA protein captured reached ~2,000 RU. Compounds were analysed using the running buffer containing 5% DMSO. Data were corrected by using the anti-PA antibody immobilised sensor chip as a control.

### EC_50_ and CC_50_ evaluations in CV assays

The anti-influenza virus activities of compounds were evaluated as described previously^48^ with some modifications. To evaluate the anti-influenza virus activities, MDCK cells were seeded into 96-well plates at a density of 3.0 × 10^4^ cells/well in 100 μL of MEM containing 5% FBS, and then incubated overnight. Cells were washed with MEM vitamin and 100 μL of the serially diluted compound was then added. Cells were subsequently infected without (determination of CC_50_) or with (determination of EC_50_) 100 μL of virus solution in MEM vitamin equivalent to 100 TCID_50_ for type A viruses or 30 TCID_50_ for B/Lee/40. The culture plates were incubated at 37 °C for 48 h. After incubation, cells were fixed with 70% EtOH and stained with 0.5% CV. After washing with water and air drying, the absorbance was measured at 560 nm using an Infinite M200 pro plate reader (Tecan Japan Co. Ltd, Kanagawa, Japan). To determine the yield of influenza virus, the supernatant from each well was collected and the virus yield was determined using the TCID_50_ assay. EC_50_ and CC_50_ values were calculated from the dose-response curve by linear regression analysis. The selectivity index was calculated by the formula: CC_50_/EC_50_^50^.

### WST-1 assay

The WST-1 assay was performed as previously described^29^ with some modifications. Briefly, MDCK cells were seeded in 24-well plates at a density of 1.8 × 10^5^ cells/well in MEM containing 5% FBS and incubated overnight. Cells were infected with the influenza virus in the presence of PA-49 and the culture plates were then incubated at 37 °C for 46 h. The medium was replaced with 0.6 mL of MEM vitamin containing 15 μL of Cell Proliferation Reagent WST-1 (Roche) and then incubated at 37 °C for 1 h. The absorbance was measured at 450–650 nm using the plate reader. The viability of cells was determined as described above. The plates were subsequently fixed and stained with CV as described above.

### Statistical analysis

The results were expressed as the mean ± SD based on three independent experiments. The Student’s *t*-test was performed using GraphPad Prism software (Version 5.0.4, GraphPad Software, Inc, CA) to detect significant differences between the test samples and controls. *P*-values less than 0.05 were considered to indicate significant differences.

## Electronic supplementary material

supplementary info
