# Supplementary material for: Structure-based drug discovery for combating influenza virus by targeting the PA–PB1 interaction
Source: Sci Rep. 2017 Aug 25;7:9500. doi: 10.1038/s41598-017-10021-w (PMC5573363; doi:10.1038/s41598-017-10021-w)
Supplement: Supplementary file 1 — supplementary info [file 41598_2017_10021_MOESM1_ESM.pdf]

Supplementary Information

Structure-based drug discovery for combating influenza virus by targeting the PA–PB1 interaction

Ken Watanabe[1*], Takeshi Ishikawa[1], Hiroki Otaki[1], Satoshi Mizuta[1], Tsuyoshi Hamada[2], Takehiro Nakagaki[1], Daisuke Ishibashi[1], Shuzo Urata[3], Jiro Yasuda[3], Yoshimasa Tanaka[1]& Noriyuki Nishida[1,2]

[1]Department of Molecular Microbiology and Immunology, Graduate School of Biomedical Sciences, Nagasaki University, 1-12-4 Sakamoto, Nagasaki, 852-8523, Japan

[2]Nagasaki Advanced Computing Center, Nagasaki University, 1-14 Bunkyo-machi, Nagasaki, 852-8521, Japan

[3] Department of Emerging Infectious Diseases, Institute of Tropical Medicine, Nagasaki University, 1-12-4 Sakamoto, Nagasaki, 852-8523, Japan

*Correspondence and requests for materials should be addressed to K.W. (email: kenw@nagasaki-u.ac.jp)

Supplementary Methods

Hit compounds

IUPAC name and molecular weight of hit compounds are as follows: PA-37 (BAS02158116, 4-{2-chloro-4-[3-(methoxycarbonyl)-2-methyl-5-oxo-1H,4H,5H-indeno[1,2-b]pyridin-4-yl]phenoxymethyl}benzoic acid, molecular weight = 515.94), PA-49 (BAS04419780, 3-({benzyl[(4-fluorophenyl)methyl]amino}[1-(2-methylbutan-2-yl)-1H-1,2,3,4-tetrazol-5-yl]methyl)-8-methyl-1,2-dihydroquinolin-2-one, molecular weight = 524.63), PA-58 (BAS01519074, 19-isopropyl-5,9-dimethyl-15-(4-methylphenyl)-14,16-dioxo-15-azapentacyclo[10.5.2.0~1,10~.0~4,9~.0~13,17~]nonadec-18-ene-5-carboxylic acid, molecular weight = 489.65) and PA-107 (ASN10813896, 3-[(3-fluorophenyl)methyl]-5-[1-(4-phenylbenzoyl)piperidin-4-yl]-3H-[1,2,3]triazolo[4,5-d]pyrimidin-7-ol, molecular weight = 508.55).

Cells, viruses and antibodies

Vero 76 cells and Huh-7 cells were obtained from the National Institute of Biomedical Innovation (JCRB9007 and JCRB0403). SW13 cells were obtained from Dr Roger Hewson (Public Health England, UK). HEK293T cells were obtained from Dr Hideki Hayashi (Nagasaki University, Japan). Vero 76, Huh-7,SW13 and HEK293T cells were maintained in Dulbecco's modified Eagle's medium containing 10 % FBS at 37°C in an atmosphere of 5% CO_2. VSV was obtained from Dr Hiroshi Kida (Hokkaido University, Japan) and propagated in Vero 76 cells. HAZV was obtained from Dr Roger Hewson and propagated in SW13 cells. SFTSV was obtained from Dr Ken Maeda (Yamaguchi University, Japan) and propagated in Vero 76 cells. The Armstrong strain of recombinant LCMV was rescued using the reverse genetics method[1] and propagated in Vero 76 cells. All virus stocks were stored at −80°C. Anti-LCMV NP antibody (clone VL-4) was obtained from BioXCell, (West Lebanon, NH).

Antiviral tests using VSV, HAZV, SFTSV and LCMV

Huh-7 cells were seeded in 96-well plates (3×10^4 cells/well) one day before infection. The next day, the virus was infected at an MOI of 0.1 and incubated for 90 min under 5% CO_2 at 37°C. After incubation, the culture medium was replaced with fresh medium containing a specific concentration of the compound. At 24 hpi, the culture supernatant was collected, centrifuged (1,500 × *g*, 5 min, 4 °C) to clarify the cell debris, and the collected supernatant was used for viral titration.

Virus titration for VSV, HAZV, SFTSV and LCMV

VSV titration was performed using a plaque assay. Briefly, Vero 76 cells were seeded in 24-well plates (1×10^5/well). On the next day, a 10 times dilution of VSV was prepared in 2% FBS in Opti-MEM (Gibco). The culture medium for Vero 76 cells was replaced with diluted VSV for 90 min and then replaced with 1% agarose in MEM including 1% P/S. At 24 hpi, the infected cells were fixed with 4% paraformaldehyde (PFA) before removing the agarose gel and staining with CV. Plaques were counted and the viral titre was calculated as pfu/mL. HAZV was also titrated using a plaque assay. Briefly, SW13 cells were seeded in 24-well plates (1.5×10^5/well). On the next day, a 10 times dilution of HAZV was prepared in 2% FBS in Opti-MEM. The culture medium for SW13 cells was replaced with diluted HAZV for 90 min and then replaced with 0.5% agarose in MEM including 1% P/S. At four days post-infection, the infected cells were fixed with 4% PFA, before removing the agarose gel and staining with CV. Plaques were counted and the viral titre was calculated as pfu/mL. To titrate SFTSV, Vero 76 cells were seeded one day before infection. The cells were infected with a 10-times virus dilution and incubated for 16 h at 37°C under 5% CO_2. The cells were fixed with 4% PFA for 30 min and incubated with PBS-T for 1 h at room temperature. Blocking was performed overnight at 4°C with 10% FBS/dilution buffer (3% BSA and 0.3% Triton-X100/PBS (–)). SFTSV N protein was detected using anti-SFTSV N antibody, followed by anti-rabbit IgG-FITC antibody (Abcam Plc, Cambridge UK; ab6009). N-positive cells were counted and normalised as fluorescent focus units (FFU)/mL. To titrate LCMV, Vero 76 cells were seeded one day before infection. The cells were infected with a 10 times virus dilution and incubated for 16 h at 37°C under 5% CO_2. The cells were fixed with 4% PFA for 30 min at room temperature. Blocking was performed at 4°C for 1 h with 10% FBS/dilution buffer. Anti-LCMV NP antibody (BioXCell; VL-4) was used as the first antibody, and after incubation for 2 h at room temperature the cells were washed with PBS (-) before reacting with the second antibody (488-Goat anti-rat IgG antibody (Abcam; ab150157)) for 2 h at room temperature. NP-positive cells were counted and normalised as FFU/mL. All virus stocks were stored at −80°C.

Cytotoxicity assay

The effect of the test compound on Huh-7 cell viability was assessed using the CellTiter-Glo luminescent cell viability assay (Promega). The procedure described above for the antiviral test was also used for the assay without virus infection. The assay was performed according

to the manufacturer's recommendations and readings were obtained using a luminometre (Tristar LB941, BERTHOLD).

Fig. S1

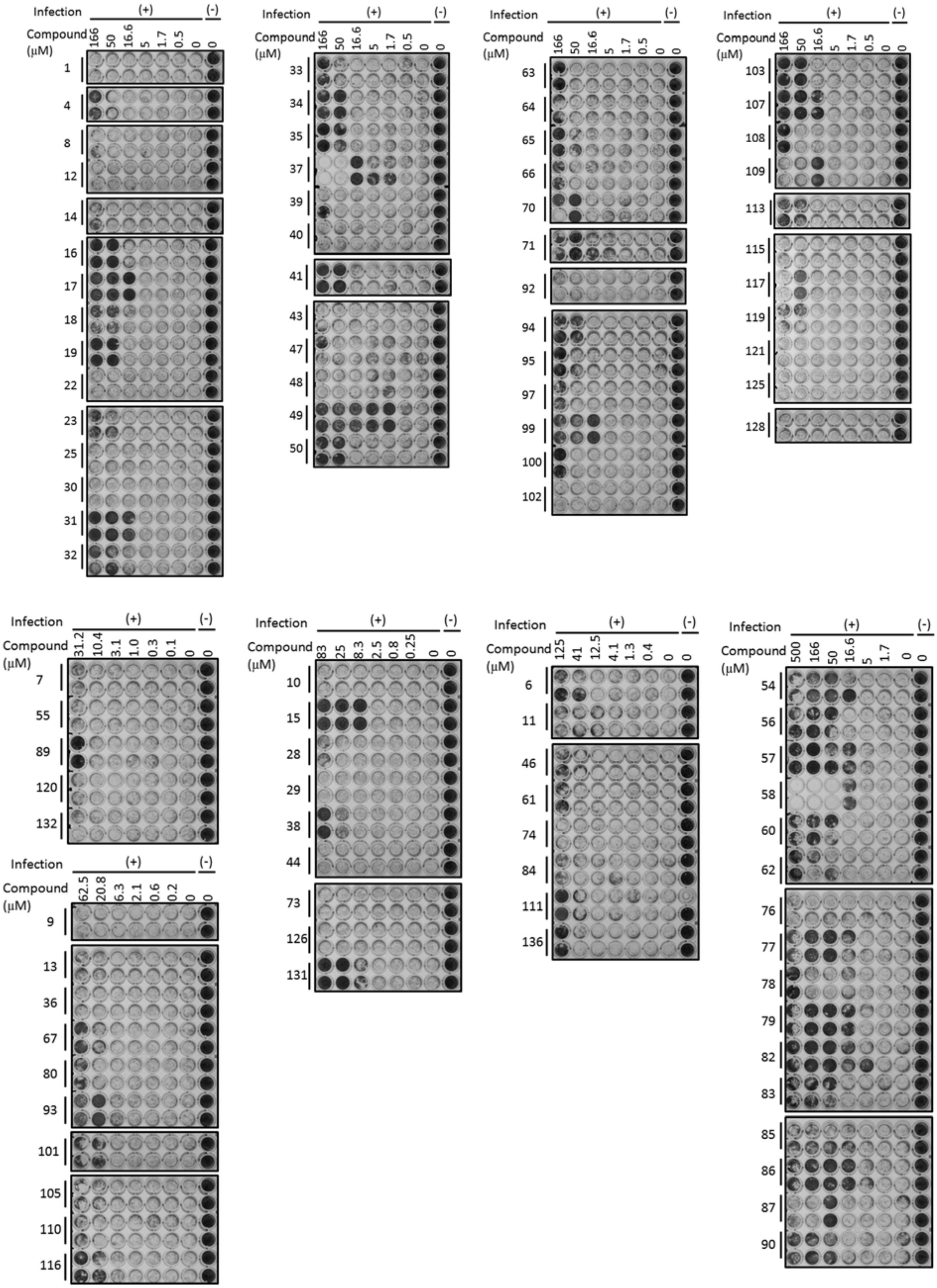
Infection (+) (-)
Compound (μM) 166 50 16.6 5 1.7 0.5 0 0
1
4
8
12
14
16
17
18
19
22
23
25
30
31
32
33
34
35
37
39
40
41
43
47
48
49
50
63
64
65
66
70
71
92
94
95
97
99
100
102
103
107
108
109
113
115
117
119
121
125
128
Compound (μM) 31.2 10.4 3.1 1.0 0.3 0.1 0 0
7
55
89
120
132
Compound (μM) 62.5 20.8 6.3 2.1 0.6 0.2 0 0
9
13
36
67
80
93
101
105
110
116
Compound (μM) 83 25 8.3 2.5 0.8 0.25 0 0
10
15
28
29
38
44
73
126
131
Compound (μM) 125 41 12.5 4.1 1.3 0.4 0 0
6
11
46
61
74
84
111
136
Compound (μM) 500 166 50 16.6 5 1.7 0 0
54
56
57
58
60
62
76
77
78
79
82
83
85
86
87
90

Supplementary figure S1. Cell-based screening of anti-influenza virus compounds. Compounds selected by *in silico* screening were tested to determine their anti-influenza virus activities. Serially diluted compounds were added to MDCK cells in the presence of influenza virus and incubated for 48 h. The cells were fixed and stained with CV and the anti-influenza virus activity was estimated visually. Compounds with antiviral activities < 20 μM were considered to be positive results. Each experiment was performed in duplicate and repeated twice.

Fig. S2

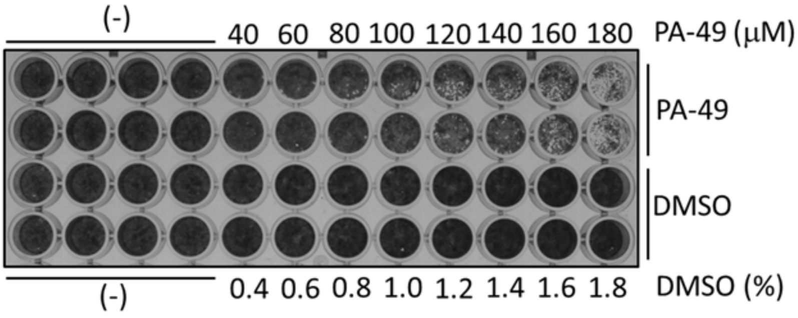

Supplementary figure S2. Evaluation of cytotoxicity of PA-49 by CV assay. MDCK cells grown in 96-well plates were treated with 40 - 180 μM of PA-49 or DMSO for 48 h. Cells were fixed and stained with CV.

Fig. S3

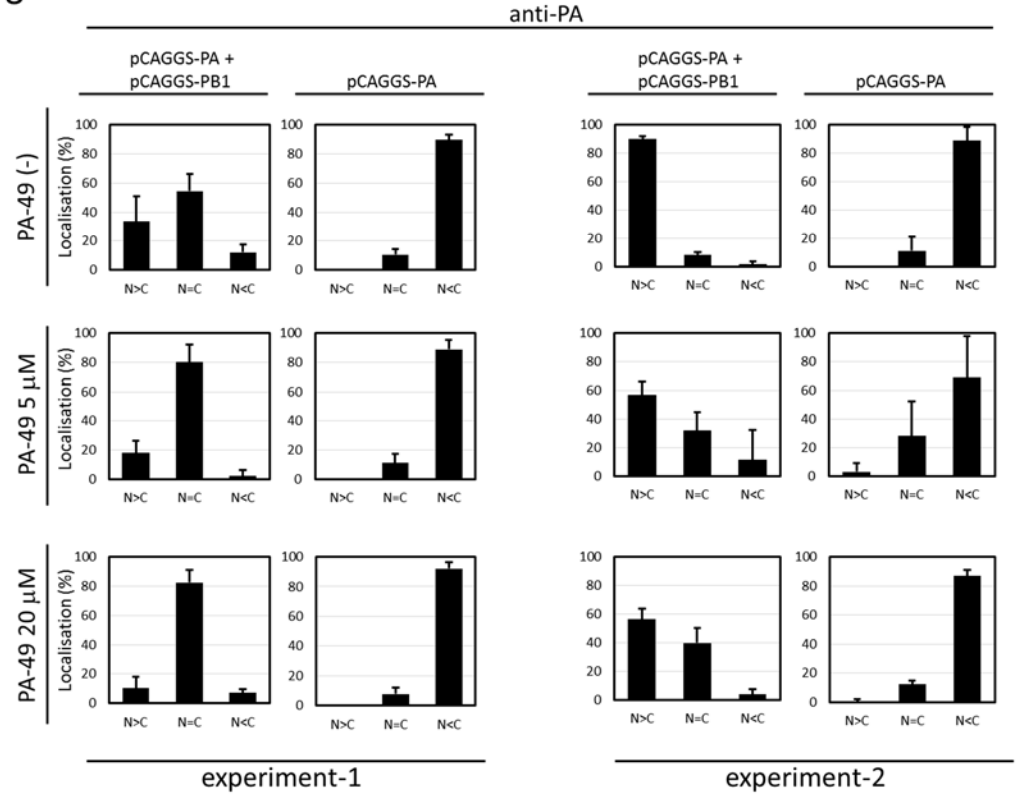

Supplementary figure S3. Dose-response effect of PA-49 on subcellular localisation of PA protein. Two additional experiments were performed as the same experimental condition in the main figure 3 except for lower PA-49 concentration (5 or 20 μM). The percentage of cells showing greater nuclear than cytoplasmic localisation (N>C), nuclear equal to cytoplasmic localisation (N=C) or cytoplasmic localisation (N<C) was determined by direct counting. The mean and standard deviation obtained from at least three different areas were shown.

Fig. S4

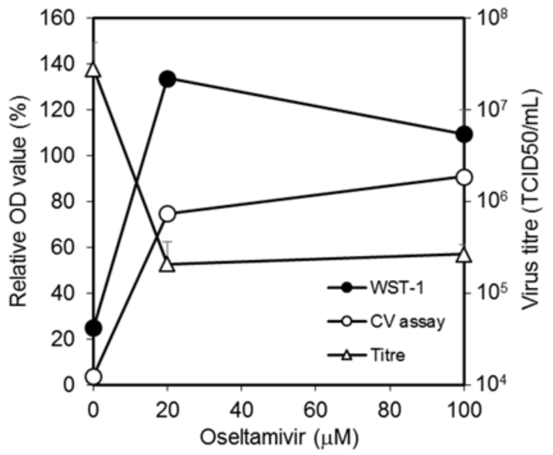

Supplementary figure S4. Anti-influenza virus activity of oseltamivir. The anti-influenza virus effects of oseltamivir were evaluated as described in the Methods section. MDCK cells grown in 24-well plates were treated with 20 or 100 μM of oseltamivir. Antiviral effects on cells were measured by the WST-1 assay (closed circles) or CV staining (open circles). Optical density (OD) values (%) are expressed relative to the percentage of cells without virus infection. Viral titres in the supernatant (open triangles) were measured by $TCID_{50}$ assays.

Fig. S5

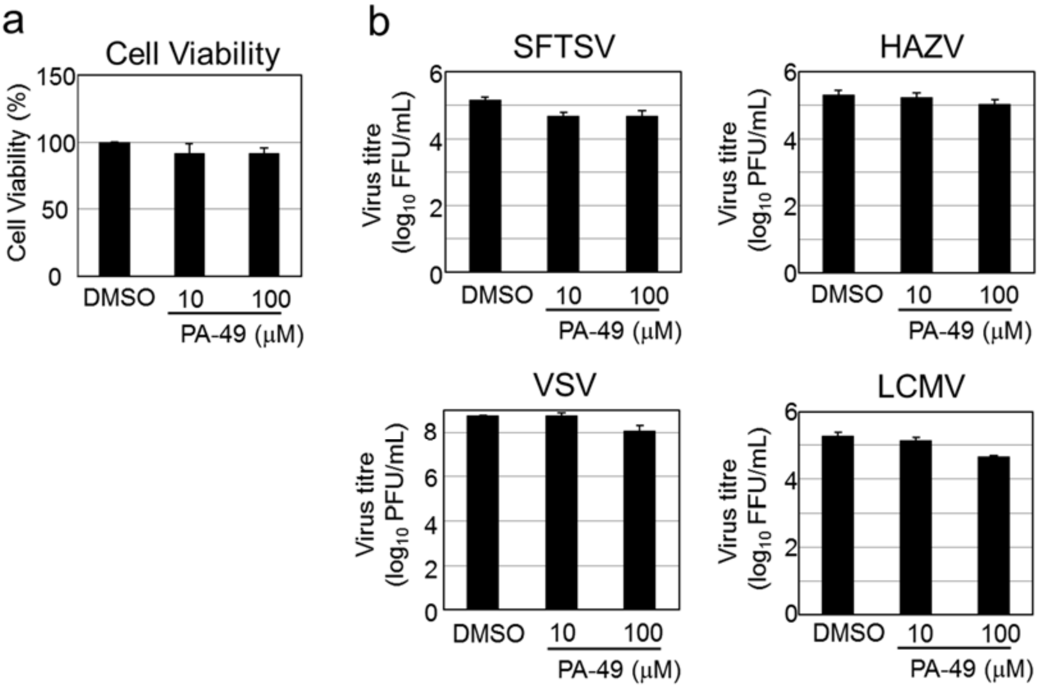

Supplementary figure S5. Effects of PA-49 against various RNA viruses. Huh-7 cells were seeded in 96-well plates (3×10^4 cells/well) one day before infection. The next day, the cells were uninfected (a) or infected (b) with SFTSV, HAZV, VSV or LCMV at an MOI of 0.1 and incubated at 37°C for 90 min. After incubation, the culture medium was replaced with fresh medium containing a specific concentration of the compound. At 24 hpi, the culture supernatant was collected and titrated. The relative cell viabilities (a) and viral titres in the supernatant are shown.

Fig. S6

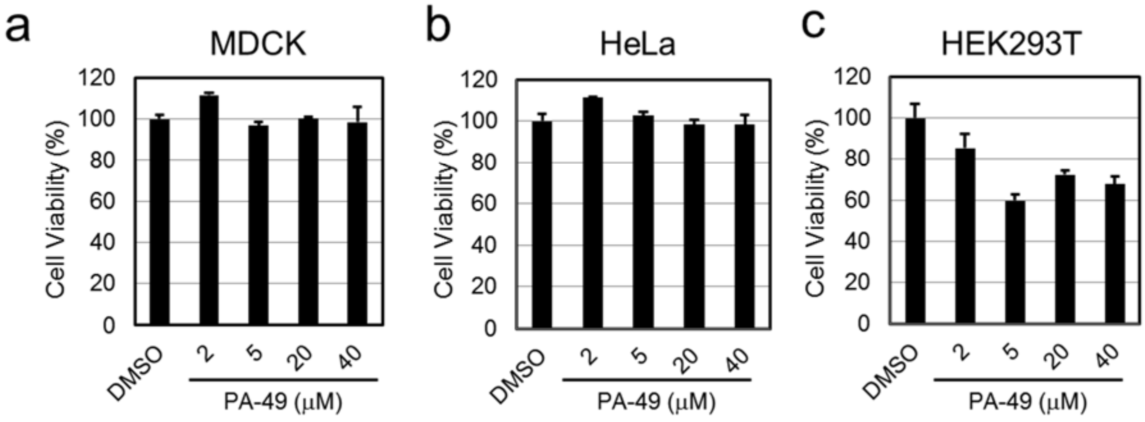

Supplementary figure S6. Evaluation of cytotoxicity of PA-49. The cytotoxic effect of PA-49 on uninfected (a) MDCK, (b) HeLa or (c) HEK293T cells were evaluated at 24 h by WST-1 assay as described in the Methods section. Average and standard deviation were obtained from duplicate measurements.

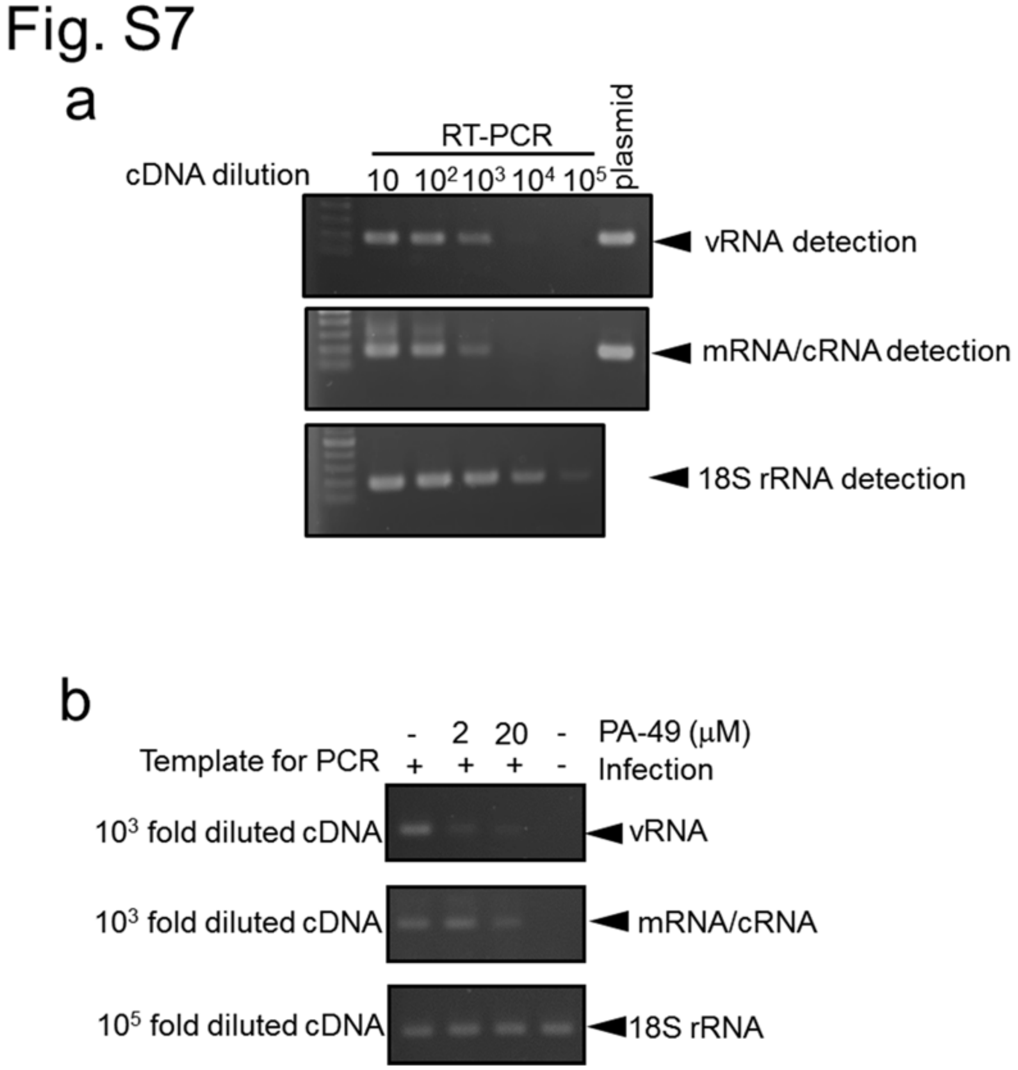

Supplementary figure S7. Effect of PA-49 on influenza virus replication. MDCK cells grown in 24-well plates (2 × 10^5 cells/well) were treated with or without A/WSN/33 virus at an MOI of 1 for 1 h. After washing, 2 and 20 μM of PA-49 was added and at 9 h after infection, the total RNA was extracted and reverse-transcribed to detect segment 5 of the viral genome RNA (vRNA; top panel). Viral messenger RNA (mRNA) and replication intermediate complementary RNA (cRNA; middle panel) were detected using different primers for reverse transcription. 18S rRNA was also amplified as a control. (a) Determination of the cDNA dilution factor. To obtain semi-quantitative results, cDNA samples were serially diluted and subjected to PCR. We used 10^3, 10^3 and 10^5-fold diluted cDNA samples for vRNA detection, mRNA/cRNA detection, and 18SrRNA detection in (b). (b) Detection of PCR products in the absence or presence of 2 and 20 μM PA-49.

Fig. S8

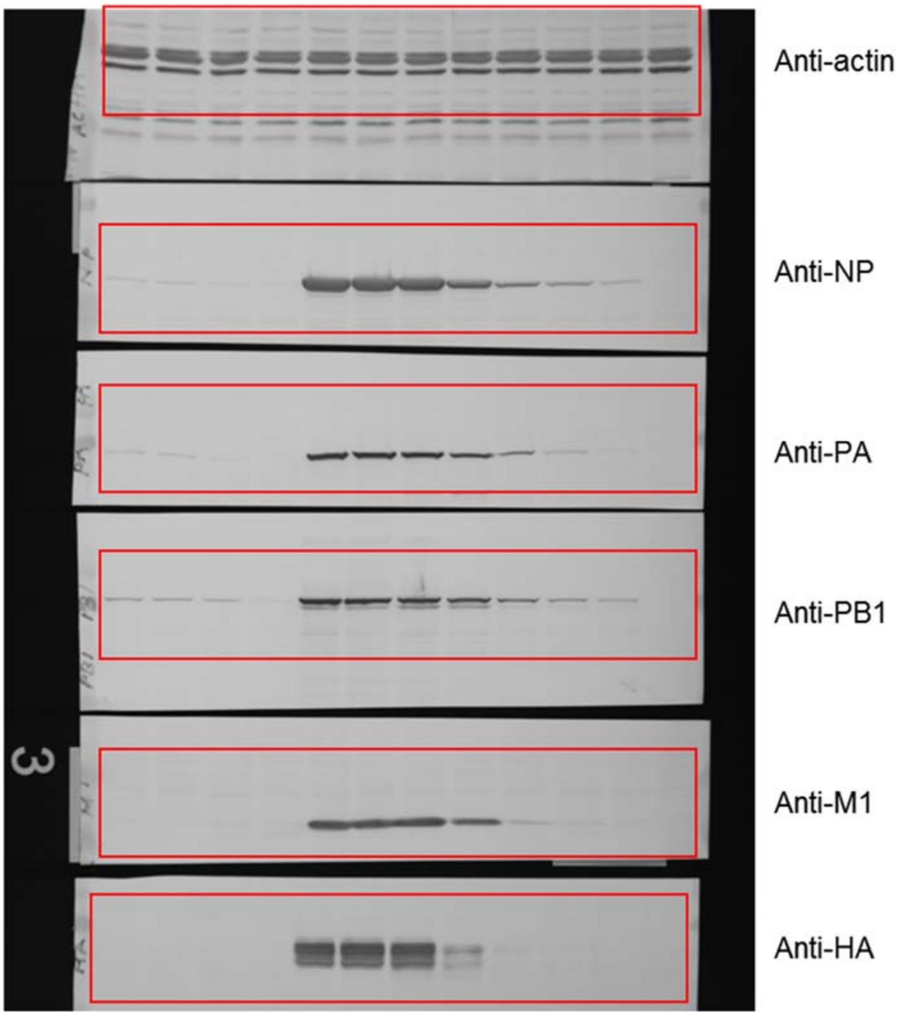

Supplementary figure S8. Original uncropped images of western blotting shown in main Figure 6.

Reference

1 Flatz, L., Bergthaler, A., de la Torre, J. C. & Pinschewer, D. D. Recovery of an arenavirus entirely from RNA polymerase I/II-driven cDNA. *Proc Natl Acad Sci U S A* **103**, 4663-4668 (2006).
